# Monocytic Tissue Transglutaminase in a Rat Model for Reversible Acute Rejection and Chronic Renal Allograft Injury

**DOI:** 10.1155/2015/429653

**Published:** 2015-04-30

**Authors:** Anna Zakrzewicz, Srebrena Atanasova, Winfried Padberg, Veronika Grau

**Affiliations:** Department of General and Thoracic Surgery, Laboratory of Experimental Surgery, Justus Liebig University Giessen, 35385 Giessen, Germany

## Abstract

Acute rejection is a major risk factor for chronic allograft injury (CAI). Blood leukocytes interacting with allograft endothelial cells during acute rejection were suggested to contribute to the still enigmatic pathogenesis of CAI. We hypothesize that tissue transglutaminase (Tgm2), a multifunctional protein and established marker of M2 macrophages, is involved in acute and chronic graft rejection. We focus on leukocytes accumulating in blood vessels of rat renal allografts (Fischer-344 to Lewis), an established model for reversible acute rejection and CAI. Monocytes in graft blood vessels overexpress Tgm2 when acute rejection peaks on day 9 after transplantation. Concomitantly, caspase-3 is activated, suggesting that Tgm2 expression is linked to apoptosis. After resolution of acute rejection on day 42, leukocytic Tgm2 levels are lower and activated caspase-3 does not differ among isografts and allografts. Cystamine was applied for 4 weeks after transplantation to inhibit extracellular transglutaminase activity, which did, however, not reduce CAI in the long run. In conclusion, this is the first report on Tgm2 expression by monocytes in vivo. Tgm2 may be involved in leukocytic apoptosis and thus in reversion of acute rejection. However, our data do not support a role of extracellular transglutaminase activity as a factor triggering CAI during self-limiting acute rejection.

## 1. Introduction

Kidney transplantation is the only curative therapy for end-stage renal disease. However, transplantation is limited not only by organ shortage but also by its unfavorable long-term success due to chronic allograft injury (CAI) [1, 2]. Chronic allograft vasculopathy (CAV), glomerulosclerosis, and interstitial fibrosis and tubular atrophy (IFTA) are the dominating histopathological findings in CAI [1, 2]. Its pathogenesis is still poorly understood, but acute rejection episodes were defined as an important risk factor and experimental data suggest that they play an important pathogenetic role [1–5].

Transplantation of kidneys in Fischer-344 (F344) to Lewis rat strain combination is a well-established experimental model for human CAI [6]. We and others thoroughly characterized a variant of this model, where immunosuppression is omitted [7–10]. This model allows investigation of a severe but reversible acute rejection episode, which peaks 9 days after allogenic transplantation and precedes the development of CAI. Acute rejection of F344 to Lewis renal allografts is characterized by a strong mononuclear infiltration of the allograft interstitium [7]. At the same time, numerous leukocytes accumulate in the arterial, capillary, and venous blood vessels [9]. After resolution of acute rejection, the number of intravascular leukocytes decreases but remains chronically elevated compared to healthy kidneys [9]. A majority of blood leukocytes in day 9 allografts are monocytes displaying a unique state of partial activation regarding cell surface antigen and cytokine expression [9]. These cells interact with graft endothelial cells and might trigger CAV, which is characterized by intimal hyperplasia. To identify factors contributing to the pathogenesis of CAI during reversible acute rejection, we compared the transcriptome of mononuclear leukocytes isolated from renal allografts to isografts 9 days after transplantation. In this gene array experiment, tissue transglutaminase (transglutaminase 2, Tgm2) was found among the genes, which were upregulated by mononuclear leukocytes accumulating in the lumina of allograft blood vessels [9]. Tgm2 might be an interesting mediator in acute and chronic allograft rejection.

Transglutaminases catalyze posttranscriptional modifications of proteins, resulting in cross-linking of proteins such as cytoskeleton or extracellular matrix (ECM), as well as in modifications via amine incorporation into proteins and via deamidation [11, 12]. These modifications can have drastic consequences including increased mechanical stability, formation of autoantigens, and profound changes in protein function [11, 13, 14]. Among the 9 transglutaminases present in humans, Tgm2 is most intensively investigated and a bewildering functional diversity is described [15]. It acts as classical Ca^2+^-dependent transglutaminase, as G-protein, as disulfide isomerase, and as protein kinase [16, 17]. In addition, it interacts with a plethora of other proteins and is involved in numerous cellular processes such as apoptosis and cell survival, phagocytosis, cell adhesion and migration, and cell signaling [13]. Tgm2 is ubiquitously expressed and localizes to virtually all compartments of the cell including the cytoplasm, the nucleus, and mitochondria. In addition, Tgm2 can be attached to the surface and is secreted into the extracellular space, where it can cross-link itself to extracellular proteins [15, 18].

We are interested in Tgm2 in the context of reversible acute rejection and subsequent chronic deterioration of renal allografts. Tgm2 might exert essential functions involved in rejection such as antigen presentation and T cell activation via dendritic cells [19], activation of the NF*κ*B pathway, and enabling the expression of proinflammatory factors, leukocyte adhesion to vascular endothelial cells, and leukocyte migration [20]. During resolution of acute rejection, its role in apoptosis, phagocytosis, and release of TGF-*β* may be of importance [21, 22]. Finally, during the development of CAI, Tgm2 might protect graft blood vessels as already evidenced in the context of atherosclerosis [23]. Alternatively, Tgm2 could contribute to vascular remodeling by increasing vascular stiffness and by promoting epithelial/endothelial to mesenchymal transition [24, 25], which was suggested to contribute to IFTA.

In this study, we investigate Tgm2 mRNA and protein expression by mononuclear blood leukocytes isolated from Lewis to Lewis isografts and F344 to Lewis allografts and by respective renal tissue Tgm2 expressions compared to activated caspase-3, a protease critically involved in apoptosis [26]. Leukocytes and grafts are studied during acute rejection on day 9 after transplantation and after resolution of acute rejection on day 42. Furthermore, allograft recipients were treated with cystamine during the first month after transplantation to assess the role of early extracellular transglutaminase activity in the pathogenesis of CAI.

## 2. Materials and Methods

### 2.1. Animal Experiments

Male Lewis (RT1^1^) (Janvier, St. Berthevin, France) and F344 (RT1^lv1^) (Harlan Winkelmann, Borchen, Germany) rats weighing 270–300 g were used for transplantation. Animals were kept under conventional conditions and received humane care according to the German Law on the Protection of Animals and the “Principles of Laboratory Animal Care” formulated by the National Society for Medical Research as well as the NIH “Guide for the Care and Use of Laboratory Animals.” Experiments were approved by the local authorities (permit number GI 20/10 number 23/2008; GI 20/27 number 51/2010).

Kidneys were transplanted orthotopically to totally nephrectomized Lewis recipients as described previously [27], except that the ureter was anastomosed end to end. For allogenic transplantation, F344 rats and, for isogenic transplantation, Lewis rats served as donors. Lewis rats were used as recipients or untreated controls. Recipients obtained i.p. 30 *μ*g ampicillin after surgery (Ratiopharm, Ulm, Germany); no immunosuppressant was given. Total ischemic times remained below 30 min. On day 9 or 42 after transplantation, graft recipients were anesthetized by i.p. application of 60 mg/kg sodium pentobarbital (Narcoren, Merial, Hallbergmoos, Germany) and grafts were removed, cut in small pieces, and snap frozen in liquid nitrogen until use.

Allograft recipients were treated with the transglutaminase inhibitor cystamine dihydrochloride (Sigma-Aldrich, Taufkirchen, Germany) for 28 days starting at the day of transplantation. Cystamine was diluted in sterile saline and delivered continuously using subcutaneously implanted osmotic minipumps (2ML4 Alzet, Cupertino, CA) at a concentration of 12 mg per day. Control animals received saline alone. Animals were sacrificed 12 weeks after transplantation; grafts were removed, fixed in 4% buffered paraformaldehyde, and embedded in paraffin.

### 2.2. Renal Function

To study renal function, urine was collected for 24 h from animals treated with cystamine or placebo. Creatinine was determined in urine and blood plasma and creatinine clearance was calculated.

### 2.3. Isolation of Mononuclear Leukocytes from the Renal Vasculature

Isolation of mononuclear blood cells was described previously in detail [28, 29]. Briefly, recipients were anaesthetized and heparinized by i.v. injection of 200 U of heparin (Liquemin N 5000, Roche, Basel, Switzerland), and kidneys were intensively perfused with cold Ca^2+^- and Mg^2+^-free phosphate buffered saline (PAA, Pasching, Austria), supplemented with 2.7 mM EDTA and 0.1% bovine serum albumin (Serva, Heidelberg, Germany). To deplete erythrocytes and granulocytes, perfusates were purified by Percoll density centrifugation as described previously [28]. Mononuclear cells were counted and stored under liquid nitrogen until use.

### 2.4. RNA Isolation and cDNA Synthesis

Total RNA was isolated from 30 mg kidney tissue or from 5 × 10^6^ mononuclear leukocytes harvested from the blood vessels of control kidneys, isografts, or allografts on day 9 or 42 after transplantation. RNA extraction was performed using the RNeasy Mini Kit (Qiagen, Hilden, Germany) according to the instructions of the supplier. One *μ*g of total RNA was reversely transcribed using the M-MLV H^−^ reverse transcriptase and 1 *μ*g of random hexamer primers (Promega, Mannheim, Germany). The reaction was carried out at 40°C for 1 h.

### 2.5. Real-Time RT-PCR

Real-time RT-PCR was used to assess the mRNA gene expression of Tgm2. Reactions were performed in an ABI 7900 Sequence Detection System (Applied Biosystems, Foster City, CA) using Platinum SYBR green qPCR Super Mix-UDG (Invitrogen, Karlsruhe, Germany). Each sample was assessed in duplicate. The relative gene expression values were calculated by the equation 2^ΔCt^, where ΔCt is the difference in Ct values between the porphobilinogen deaminase (PBGD) housekeeping gene and Tgm2. Data for each experimental group are expressed in relation to the expression in the healthy control, which was set to 1 arbitrary unit. All primers for real-time RT-PCR were synthesized by MWG Biotech (Ebersberg, Germany). Primer sequences are as follows: for Tgm2, 5′-CCA GCG TGG ACA GAC TTA CA-3′ (sense), 5′-CTG CTC CAC ATC GTC AGA CA-3′(antisense) and for PBGD, 5′-GGC GCA GCT ACA GAG AAA GT-3′(sense), 5′-AGC CAG GAT AAT GGC ACT GA-3′(antisense). PCR conditions included denaturation for 5 min at 95°C, followed by 45 cycles of 20 s at 95°C, 20 s at 60°C, and 10 s at 72°C. To confirm the production of a single amplicon with the expected molecular mass, the PCR product was analyzed by electrophoresis on agarose gels. In addition, each product was sequenced (Seqlab, Göttingen, Germany) to confirm the specificity of the PCR. A negative control, where cDNA was omitted, was included in every run. In negative controls, no product was obtained.

### 2.6. Protein Extraction and Immunoblotting

Protein extracts from tissue or from mononuclear cells were prepared as described previously [30]. Protein concentrations were determined using Micro BCA protein assay kit (Pierce Biotechnology, Rockford, IL). Equal amounts of protein (40 *μ*g of tissue extract or 8 *μ*g of cellular extract) were resolved on 8% or 15% reducing SDS-polyacrylamide gels and transferred onto polyvinylidene difluoride membranes (Millipore, Billerica, MA). Membranes were blocked in 1x Roti-Block solution (Roth, Karlsruhe, Germany) diluted in 50 mM Tris-HCl, pH 7.6, and 0.9% NaCl for 60 min at RT and then incubated overnight at 4°C with mouse monoclonal antibodies (mAbs) to Tgm2 (clone TG100) (Thermo Fisher Scientific, Fremont, CA) diluted 1 : 4000 or 1 : 6000 for cellular extract or kidney tissue, respectively. Optionally, membranes were incubated overnight with rabbit anti-active caspase-3 Abs (Abcam, Cambridge, UK) diluted 1 : 1000 in Roti-Block solution. To ensure equal protein loading, membranes were blocked with 5% milk and incubated with mAbs to GAPDH (Novus Biologicals, Littleton, CO), 1 : 20000. Bound antibodies were visualized with horseradish peroxidase-conjugated secondary Abs (Dako, Glostrup, Denmark), 1 : 5000, using the chemiluminescent reagent Lumi-Light Western Blotting Substrate (Roche, Mannheim, Germany). Densitometric analyses were performed using a digital gel documentation system (Biozym, Hessisch Oldendorf, Germany). All data of individual samples were divided by the values obtained for GAPDH on the same blot. The mean of the Tgm2/GAPDH or active caspase-3/GAPDH ratio of the controls was set to 1 arbitrary unit and each individual value including control values was calculated accordingly.

### 2.7. Orcein Staining and Quantification of Arterial Remodeling

Histological sections were stained with acidic orcein 12 weeks after transplantation to visualize the internal and external elastic lamina of arteries and to evaluate a possible effect of cystamine on arterial remodelling. At least 10 arteries of the muscular type were investigated per section. To estimate the relative thickness of arterial media and intima, the ratio of the total vessel diameter, including media and intima, and the diameter of the lumen of the artery were determined. Additionally, the percentage of arteries exhibiting intimal hyperplasia was analyzed.

### 2.8. Azocarmine/Aniline Blue Staining and Evaluation of the Thickness of the Tunica Adventitia

Histological sections were stained with azocarmine/aniline blue (Azan) 12 weeks after transplantation and Tunica adventitia was measured. To estimate relative thickness of the Tunica adventitia, the diameter of outer arterial diameter (including Tunica adventitia, media, intima, and lumen) was determined and divided by diameter of inner arterial diameter (including media, intima, and lumen). At least 10 arteries of muscular type were investigated per section.

### 2.9. Immunohistochemistry

Kidneys were fixed in 4% buffered paraformaldehyde and embedded in paraffin. Sections of 6 *μ*m were dewaxed and rehydrated. To unmask Tgm2 immunoreactivity in the fixed tissue, antigen retrieval was performed in 0.01 M sodium citrate buffer (pH 6.0) for 15 min at 120°C in a steamer. Sections were pretreated with protease XIV (Sigma-Aldrich) for 15 min at RT for detection of CD163 or active caspase-3. Endogenous peroxidase activity was inactivated with 1% H_2_O_2_ in PBS for 30 min. After washing in PBS, pH 7.2, the paraffin sections were incubated for 30 min at RT with PBS supplemented with 1% BSA and 0.1% NaN_3_. Thereafter, mouse mAbs to Tgm2 diluted 1 : 1500 or ED2 (anti-CD163, Serotec, Oxford, UK) Abs diluted 1 : 200 or polyclonal rabbit Abs to active caspase-3 (Abcam) diluted 1 : 100 in PBS supplemented with 1% BSA (Serva) were applied and incubated at 4°C overnight. Bound, primary Tgm2 Abs were detected using rabbit anti-mouse immunoglobulin and goat anti-rabbit HRP labeled Abs (Dako) and 3,3′-diaminobenzidine (DAB) (Sigma-Aldrich). To amplify the signal for ED2 Abs, additionally, anti-rabbit EnVision peroxidase system (Dako) was applied according to the manufacturer's instructions. To detect Abs bound to active caspase-3, anti-rabbit EnVision peroxidase system (Dako) was used. Sections were weakly stained with hemalum and coverslipped with Pertex (Medite, Burgdorf, Germany).

To detect Tgm2-positive monocytes/macrophages, sections were first stained with mAbs to Tgm2 as described, followed by protease XIV treatment for 15 min at RT and overnight incubation at 4°C with mAb ED1 directed to a CD68-like antigen (Serotec) diluted 1 : 500 in PBS supplemented with 1% BSA and 0.1% NaN_3_. To detect bound primary Abs, rabbit anti-mouse immunoglobulin and alkaline phosphatase anti-alkaline phosphatase (APAAP) (both from Dako) were used in combination with Fast Blue (Sigma-Aldrich) as chromogen. Sections were coverslipped with Glycergel (Dako).

As a control, immunohistochemical staining was performed in the absence of primary Abs. This resulted in the absence of staining. In addition, for double-staining each primary Ab was used alone. Sections were evaluated with an Olympus BX51 microscope and ANALYSIS software (Olympus, Hamburg, Germany).

### 2.10. Statistics

Data were analyzed, where applicable, by nonparametric Kruskal-Wallis test followed by the Mann-Whitney rank sum test using SPSS software (SPSS software, Munich, Germany). *p* values below 0.05 were indicated.

## 3. Results

### 3.1. Accumulation of Leukocytes in Allograft Blood Vessels

**Figure 1:**
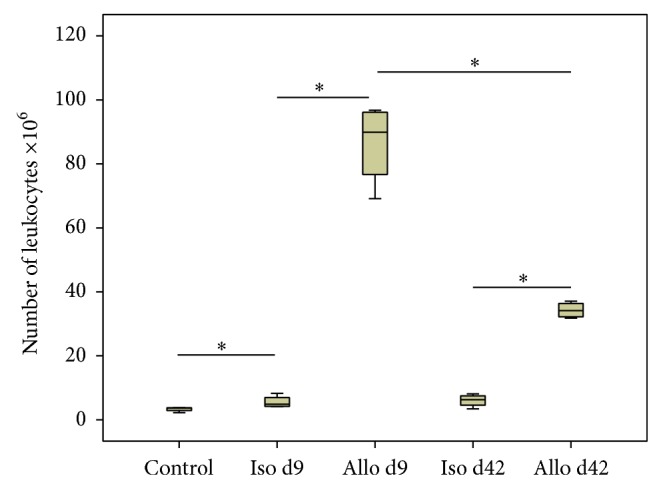
Accumulation of mononuclear leukocytes in graft blood vessels. The number of leukocytes obtained by vascular perfusion of untreated Lewis kidneys (control), isografts (Iso), and allografts (Allo) on day 9 (d9) and day 42 (d42) after transplantation (*n* = 4 per group is depicted). Box plots indicate median and percentiles 0, 25, 75, and 100. ^∗^ *p* ≤ 0.05.

Renal transplantation in the F344 to Lewis rat strain combination results in accumulation of mononuclear leukocytes in allograft blood vessels [9, 31]. To isolate these cells, kidneys were intensively perfused on days 9 and 42 after transplantation. In agreement with the previously published data, we observed a strong increase in the number of mononuclear leukocytes accumulating in the vasculature of allografts on day 9 compared to isografts. Furthermore, the number of intravascular leukocytes was reduced on day 42; however, it remained still increased compared to day 42 isografts. The difference in cell number was also observed between perfusates from control kidneys and isografts on days 9 and 42 (Figure 1). The cellular composition of intravascular leukocytes obtained by perfusion on day 9 and day 42 was published before [9, 31].

### 3.2. Tgm2 mRNA Expression Revealed by Real-Time RT-PCR

**Figure 2:**
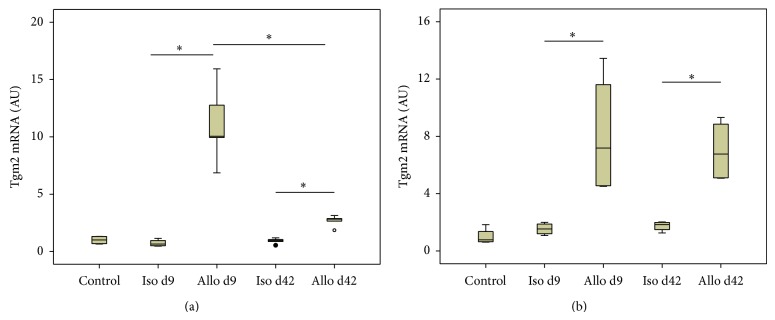
Tgm2 mRNA expression. Changes in the expression levels of Tgm2 were assessed by real-time RT-PCR in intravascular leukocytes isolated from control kidneys, renal isografts (Iso), and allografts (Allo) (a) and in total tissue (b) from control and iso- and allografts on day 9 (d9) and day 42 (d42) after transplantation. Data are expressed as arbitrary units (AU), which are normalized to one unit in controls (*n* = 4 per group). Box plots indicate median and percentiles 0, 25, 75, and 100. ^∗^ *p* ≤ 0.05.

The mRNA expression of Tgm2 was analyzed by real-time RT-PCR in mononuclear leukocytes isolated from renal blood vessels of control animals and isogenic and allogenic renal transplants. Real-time RT-PCR led to a single amplicon of the expected molecular mass in all samples analyzed. In negative controls, where cDNA was omitted, no product was detected (data not shown). Interestingly, an elevated level of Tgm2 mRNA was detected in cells collected from allografts on day 9, as well as on day 42 after transplantation, when compared to the respective isografts (Figure 2(a)).

Tgm2 mRNA expression was also increased in day 9 and day 42 allograft tissue compared to respective isografts. In contrast to graft blood leukocytes, where the expression was stronger on day 9 compared to day 42, Tgm2 tissue expression did not differ between day 9 and day 42 (Figure 2(b)).

### 3.3. Tgm2 Protein Expression Revealed by Immunoblotting

**Figure 3:**
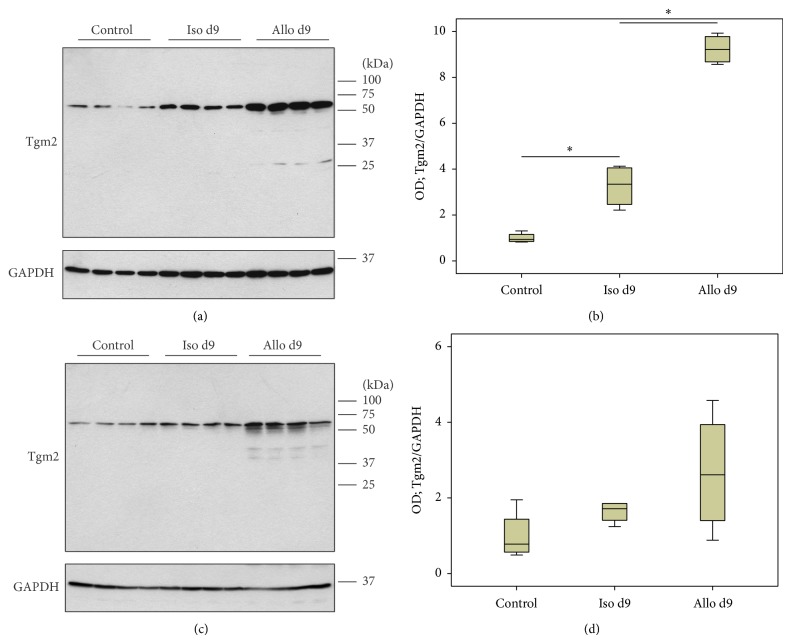
Tgm2 protein expression. Changes in the expression levels of Tgm2 were investigated by immunoblotting of lysates of mononuclear intravascular leukocytes isolated from control animals isogenic (Iso) and allogenic (Allo) renal transplants (a) as well as total control and graft tissue lysates (c) on day 9 after transplantation. Detection of the house-keeping enzyme GAPDH was performed for normalization. Densitometric quantifications of the bands are depicted in (b) and (d) for perfusates and kidney tissues, respectively (*n* = 4 per group). Box plots indicate median and percentiles 0, 25, 75, and 100. ^∗^ *p* ≤ 0.05. OD: optical density.

**Figure 4:**
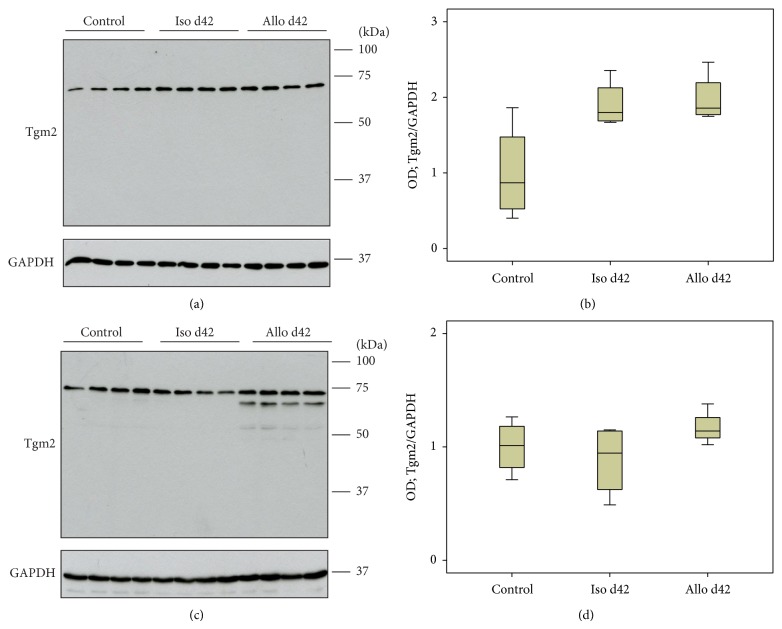
Tgm2 protein expression. Changes in the expression levels of Tgm2 were investigated by immunoblotting of lysates of mononuclear intravascular leukocytes isolated from control animals isogenic (Iso) and allogenic (Allo) renal transplants (a) as well as total control and graft tissue lysates (c) on day 42 after transplantation. Detection of the house-keeping enzyme GAPDH was performed for normalization. Densitometric quantifications of the bands are depicted for perfusates (b) and kidney tissues (d) (*n* = 4 per group). All bands detected by antibodies to Tgm2 including the additional low molecular weight bands seen in tissue samples on day 42 (c) were quantified. Box plots indicate median and percentiles 0, 25, 75, and 100. ^∗^ *p* ≤ 0.05. OD: optical density.

Protein expression of Tgm2 was investigated by immunoblotting of lysates of mononuclear intravascular leukocytes isolated from control animals and isogenic or allogenic renal transplants (Figures 3(a) and 4(a)) as well as of total tissue lysates (Figures 3(c) and 4(c)). Expression of Tgm2 was detected in all groups investigated as a major band with expected molecular mass of ~70 kDa.

On day 42, an additional Tgm2-immunoreactive band appeared in tissue samples below the major band. Densitometrical analyses revealed elevated levels of Tgm2 expression in blood mononuclear leukocytes isolated from isografts on day 9 compared to cells from control kidneys. Furthermore, an elevated expression level of Tgm2 was detected in perfusates from allografts on day 9 compared to respective isograft samples (Figure 3(b)). This observation was consistent with mRNA expression levels. In contrast to the results from real-time RT-PCR, no difference in the expression of Tgm2 was detected in perfusates on day 42 after transplantation (Figure 4(b)) as well as in total kidney tissue on day 9 and day 42 after transplantation (Figures 3(d) and 4(d)).

### 3.4. Tissue Distribution of Tgm2 Immunoreactivity

**Figure 5:**
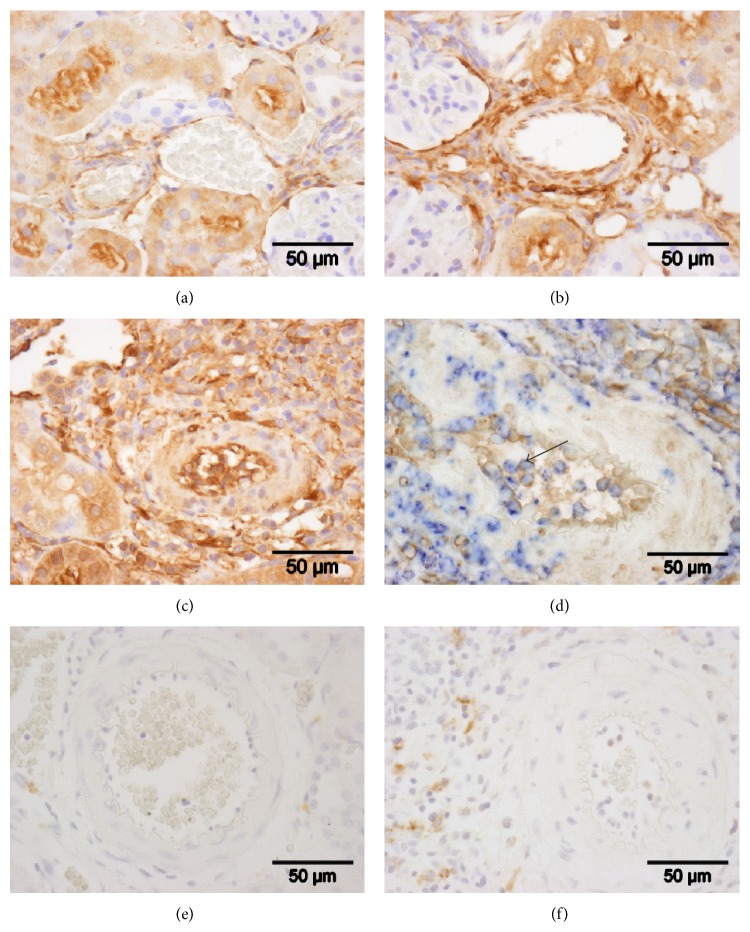
Immunohistochemical detection of Tgm2 and CD163. Immunohistochemical detection of Tgm2 was performed on paraffin sections of control kidneys (a) and isogenic (b) and allogenic (c) renal transplants on day 9 after transplantation. CD163-positive cells were stained on sections of isogenic (e) and allogenic (f) kidney grafts. Tgm2 or CD163 are labeled in brown; sections are lightly stained with hemalum. In (d), double-staining experiments using ED1 antibodies directed to a CD68-like antigen (in blue) and Tgm2 (in brown) reveal that a majority of Tgm2-positive intravascular leukocytes are monocytes. Representative sections out of four individual experiments are shown. The arrow points to a double-positive blood leukocyte.

So far, the expression of Tgm2 was analyzed in the total intravascular population of mononuclear leukocytes. To examine Tgm2 expression by graft monocytes, we performed immunohistochemical staining on paraffin sections from control kidneys, isografts, and allografts explanted on day 9 after transplantation (Figures 5(a)–5(d)). In line with previously published data describing accumulation of blood leukocytes, Tgm2-positive cells were most abundant in the vasculature of day 9 allografts (Figure 5(c)). In addition to intravascular leukocytes, blood plasma exhibited Tgm2 immunoreactivity, predominantly in allografts. To identify monocytes expressing Tgm2, double-staining experiments with mAb ED1 detecting CD68-like antigen were performed (Figure 5(d)). The majority of the blood cells positive for Tgm2 were also ED1-positive.

In isograft tissue, Tgm2 immunoreactivity was detected in the vascular endothelium of some arteries, veins, and capillaries. In the media of arteries, smooth muscle cells were immunopositive in the adventitia extracellular material and cells with a fibroblast-like shape. In the renal parenchyma, renal tubules, most probably proximal tubules, collecting ducts, and the outer layer of Bowman's capsule were stained with antibodies to Tgm2. Strong immunoreactivity was also seen in the urothelium. In renal allografts, the staining was identical to the pattern seen in isografts but it was more intense all over. In the leukocytic infiltrate, no conspicuous additional staining was detected (data not shown).

### 3.5. CD163-Positive Cells in Graft Tissue

Tgm2 was described as a marker for M2 macrophages; we investigated if graft monocytes also express the classical M2 cell surface marker CD163. To detect CD163-positive cells, immunohistochemical staining with mAb ED2 was performed in isograft and allograft tissue on day 9 after transplantation. As expected, we detected a very low number of positive cells in isografts (Figure 5(e)). In contrast, CD163-positive cells were more frequent in allografts (Figure 5(f)). In both groups analyzed, no CD163-positive cells were detected in the lumina of blood vessels.

### 3.6. Caspase-3 Activation in Blood Leukocytes, Control, and Graft Tissue

**Figure 6:**
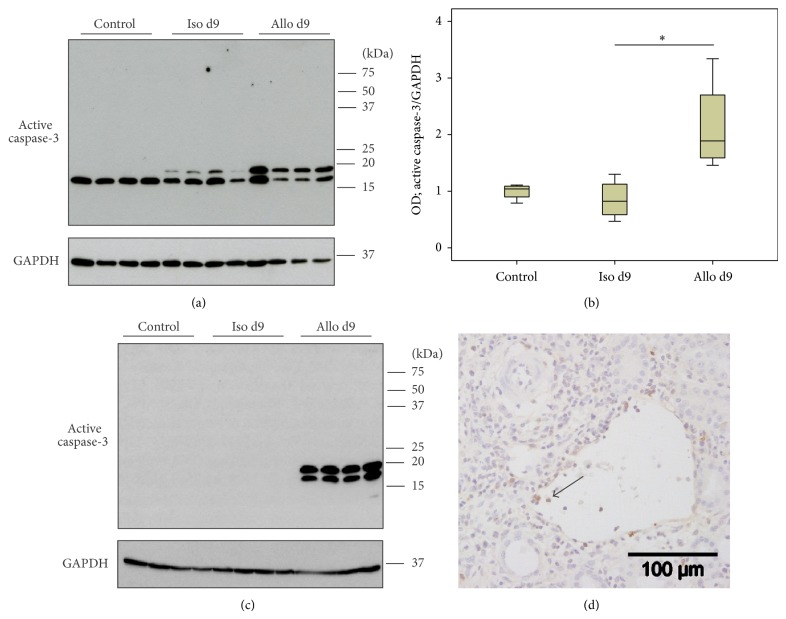
Caspase-3 activation. Changes in the activation of caspase-3 were investigated by immunoblotting of lysates of mononuclear intravascular leukocytes isolated by perfusion of control kidneys and isogenic (Iso) or allogenic (Allo) renal transplants (a) as well as total control and graft tissue lysates (c) on day 9 after transplantation. Detection of the house-keeping enzyme GAPDH was performed for normalization. Densitometric quantifications of the blot from perfusates are depicted in (b) (*n* = 4 per group). Immunohistochemical staining experiments using anti-active caspase-3 Abs (in brown) (d) reveal that active caspase-3-immunoreactive leukocytes are predominantly detected in veins of day 9 allografts. Representative sections out of four individual experiments are shown. The arrow points to a positive blood leukocyte in allograft vein. Box plots indicate median and percentiles 0, 25, 75, and 100. ^∗^ *p* ≤ 0.05. OD: optical density.

**Figure 7:**
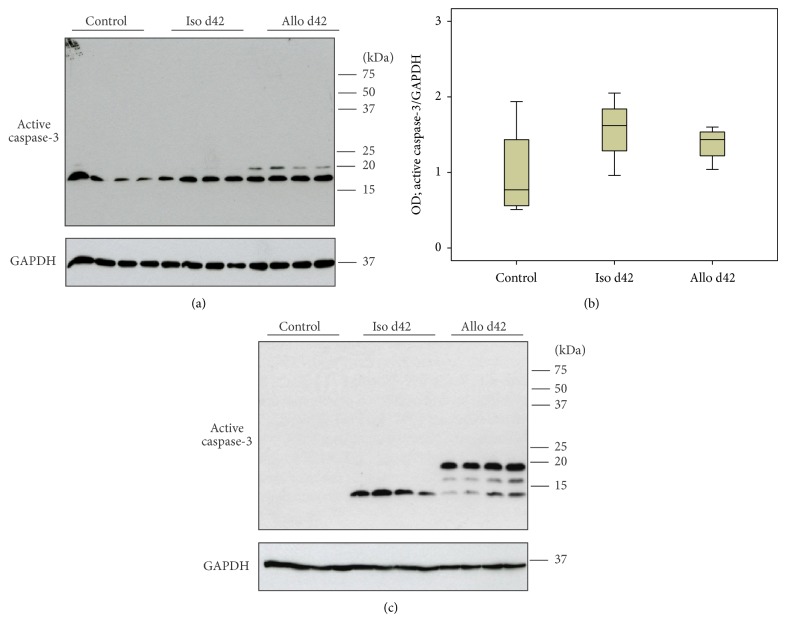
Caspase-3 activation. Changes in the activation of caspase-3 were investigated by immunoblotting of lysates of mononuclear intravascular leukocytes isolated by perfusion of control kidneys and isogenic (Iso) and allogenic (Allo) renal transplants (a) as well as total control and graft tissue lysates (c) on day 42 after transplantation. Detection of the house-keeping enzyme GAPDH was performed for normalization. Densitometric quantification of the bands from perfusate blots is depicted in (b) (*n* = 4 per group). Box plots indicate median and percentiles 0, 25, 75, and 100. ^∗^ *p* ≤ 0.05. OD: optical density.

Tgm2 is known to play a role in apoptosis. Therefore, we analyzed if caspase-3, a key protease directly involved in apoptosis, is also expressed by intravascular graft leukocytes. To examine activation of caspase-3 during reversible acute rejection, detection of active caspase-3 in renal mononuclear graft leukocytes as well as in graft tissue on days 9 and 42 after transplantation was performed by immunoblotting (Figures 6(a), 6(c), 7(a), and 7(c)). The appearance of the active p19 (~19 kDa) and p17 (~17 kDa) fragments of caspase-3 was detected. Densitometric analyses revealed increased levels of active caspase-3 in mononuclear blood leukocytes from allografts on day 9 (Figure 6(b)). Active caspase-3 was also detected in perfusates from day 42 isografts and allografts, but no difference was observed among them (Figures 7(a) and 7(b)). Also, in allograft tissue, elevated levels of active caspase-3 were measured on days 9 and 42 compared to respective isografts (Figures 6(c) and 7(c)). Furthermore, these results were corroborated by immunohistochemical staining of graft tissue on day 9 after transplantation. Active caspase-3-positive cells were detected in the graft blood vessels predominantly in allografts veins, whereas caspase-3 immunoreactivity was less prominent in leukocytes accumulating in arteries (Figure 6(d)).

### 3.7. Vascular Remodeling and Interstitial Fibrosis in Renal Allografts Treated with Cystamine

**Figure 8:**
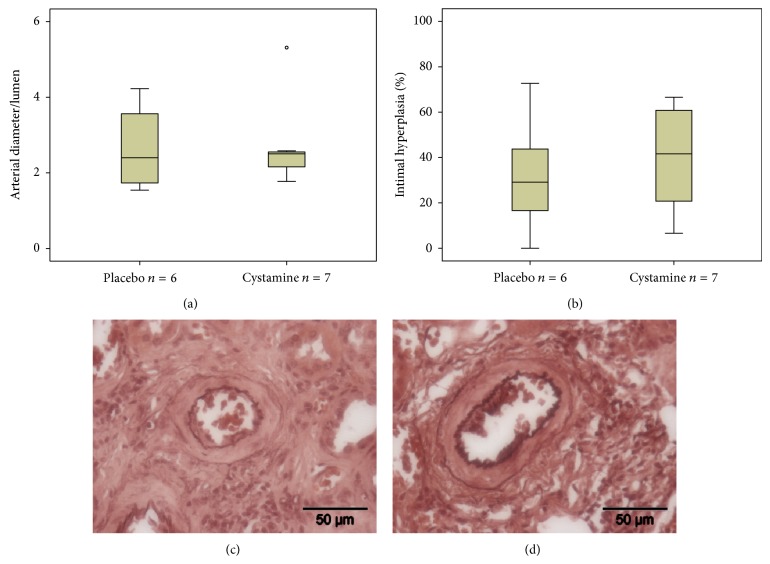
Vascular remodeling in renal allografts 12 weeks after transplantation from recipients treated with transglutaminase inhibitor cystamine or placebo. The relative arterial thickness (a) and the frequency of intimal hyperplasia (b) were investigated on histological sections from placebo- (c) or cystamine-treated (d) animals stained with orcein. Box plots indicate median and percentiles 0, 25, 75, and 100.

To examine the role of Tgm2 in the pathogenesis of CAI, renal allograft recipients were treated with cystamine, a general inhibitor of Tgm, or placebo. Creatinine clearance was monitored 4, 8, and 12 weeks after transplantation to determine renal function. No differences were observed between placebo- (3.4 ± 1.5 at 4 weeks; 5.1 ± 0.7 at 8 weeks; 3.0 ± 0.7 at 12 weeks; *n* = 6 per group; data are given as mean ± standard deviation, mL/min) and cystamine-treated animals (3.9 ± 1.2 at 4 weeks; 4.4 ± 0.6 at 8 weeks; 3.5 ± 0.5 at 12 weeks; *n* = 6 per group; data are given as mean ± standard deviation, mL/min) independent of the time point investigated. At the end of the study, at 12 weeks after transplantation, animals were sacrificed and relative thickness of arteries was quantified on sections stained with acidic orcein. No significant difference was observed between both experimental groups (Figures 8(a), 8(c), and 8(d)). Furthermore, arteries with intimal hyperplasia were detected in the sham-treated group, whereas cystamine treatment had no effect on intimal remodeling (Figures 8(b), 8(c), and 8(d)). Additionally, histological sections were stained with azocarmine/aniline blue (Azan) 12 weeks after transplantation to detect interstitial fibrosis. No difference was detected between two groups analyzed (data not shown).

## 4. Discussion

We demonstrate in this study that Tgm2 mRNA and protein are overexpressed by mononuclear leukocytes, predominantly by activated monocytes, which accumulate in the vascular bed of rat renal allografts (F344 to Lewis) on day 9 after transplantation. After resolution of acute rejection on day 42, Tgm2 mRNA levels are lower but still moderately increased compared to respective isografts, which is not reflected in increased protein levels. Tgm2 mRNA expression was also induced in graft tissue upon allogenic transplantation. On the protein level, however, no quantitative changes were detected.

We focused on the well-characterized population of mononuclear leukocytes, which accumulate in large numbers in the blood vessels of experimental allografts during acute rejection. The number of leukocytes, which were isolated from control kidneys, isografts, and allografts 9 and 42 days after transplantation by intensively perfusing renal blood vessels, nicely corroborated data published previously [9, 31]. The acute rejection episode, which peaks at around day 9 after transplantation, is of major interest for the understanding of CAI, since clinical and experimental data suggest that acute rejection is an important triggering factor [1–5]. As CAV, a hallmark of CAI, is characterized by arterial intimal hyperplasia, leukocytes interacting with allogenic endothelial cells were suggested to play a pivotal pathogenic role [9]. The number of intravascular leukocytes decreases after reversion of acute rejection in the F344 to Lewis rat strain combination but remains elevated for several weeks.

First hints regarding the overexpression of Tgm2 by mononuclear leukocytes isolated from the blood vessels of allografts on day 9 after transplantation came from gene array data [9]. These data were confirmed by real-time RT-PCR and immunoblotting using antibodies, which detected a major band of the expected molecular mass. Immunohistochemistry with the same antibodies revealed that intravascular leukocytes in renal allografts were strongly Tgm2-immunoreactive. On tissue sections double-stained with mAb ED1 directed to a CD-68-like antigen, an established marker for rat monocytes/macrophages, we identified graft monocytes as strongly Tgm2-immunopositive. It might be argued that the increase in Tgm2 expression in mononuclear blood leukocytes from day 9 allografts is due to the increase in the proportion of monocytes, which is indeed seen. However, isograft blood leukocytes contain about 50% monocytes and allografts about 70% [9], whereas Tgm2 mRNA expression levels increase by about 10-fold, suggesting that the increased proportion of monocytes is not the only explanation for the observed increase in Tgm2 mRNA levels. Interactions of monocytes with graft endothelial cells, which is a prerequisite for intravascular accumulations, may induce Tgm2 expression as described previously for monocytes adhering to endothelial cells in vitro [32]. In addition, proinflammatory cytokines produced during acute rejection [9] are known inducers of Tgm2 expression [14, 15, 33, 34].

In graft tissue, which was investigated for comparison, mRNA levels were elevated on both days 9 and 42. Tgm2 protein was detected by immunoblotting but, in contrast to Tgm2 mRNA, no significant increase in protein signals was seen. However, some weak additional bands of lower molecular mass that were only detected in allograft perfusates and tissue would be compatible with an accelerated degradation of Tgm2 in allografts. Another explanation for the discrepancy between mRNA and protein is based on reports in which Tgm2 acts as a substrate of itself and forms insoluble cross-linked complexes [15], which are not detected in conventional immunoblots. For the detection of Tgm2 covalently bound to extracellular matrix, unfixed cryostat sections should be stained in future studies [35]. In line with this idea and with our mRNA data, tissue sections of renal day 9 allografts exhibited a stronger overall staining for Tgm2 compared to respective isografts. The renal expression pattern of Tgm2 was in line with other publications on rodent and human Tgm2 expression.

Recently, Shrestha et al. reported upregulation of Tgm2 mRNA, protein, and transglutaminase activity in CAI kidneys [36]. Their model also is based on the F344 to Lewis rat strain combination but, in contrast to our model, includes a 10-day course of suboptimal immunosuppression, which interferes with early acute rejection, followed by delayed contralateral nephrectomy. The long-term outcome of both models, however, is similar. Our study and the study by these authors complement each other, as they predominantly investigate end-stage CAI, whereas we focus on the early phase posttransplantation. Shrestha et al. evidence increased mRNA levels of Tgm2 as well as increased Tgm2 immunopositivity in allograft tissue sections compared to isografts. Of note, these authors also demonstrate that, in addition to blood leukocytes investigated only in our study, renal tubules and glomeruli also overexpress Tgm2 in allografts. During the pathogenesis of CAI, urinary secretion of the transglutaminase cross-link product *ε*(*γ*-glutamyl)-lysine is increased. In end-stage CAI, this product is detected more abundantly in allografts compared to isografts [36]. From both studies, however, it is difficult to conclude if Tgm2 is a marker of CAI reflecting changes in the cellular composition of the graft or actively contributes to the pathogenesis of CAI as shown for diverse models of experimental renal fibrosis [37–40].

Tgm2 was recently described to be a reliable marker of M2 macrophages, an anti-inflammatory subpopulation of differentiated macrophages [41]. To the best of our knowledge, Tgm2 expression by blood monocytes in vivo has not been described before, although, similarly to macrophages, blood monocytes can be activated and express cytokine patterns and cell surface molecules resembling proinflammatory M1 macrophages and anti-inflammatory M2 macrophages [42]. We previously demonstrated that blood monocytes of F344 to Lewis renal allografts are activated, acquire an intermediate state of differentiation, and express both cytokines, typical for M1 and for M2 macrophages, during acute rejection [9]. We do not know, however, if this population is a mixture of M1- and M2-like monocytes or if individual monocytes are characterized by an intermediate phenotype. To further characterize these monocytes, we investigated expression of CD163, a typical M2 cell surface marker, which was not detected on graft monocytes during the peak of acute rejection. Hence, we conclude that Tgm2 can be expressed by activated monocytes but we cannot decide if, similarly to macrophages, Tgm2 expression is confined to M2-like monocytes.

The functional relevance of Tgm2 expression by M2 macrophages, however, is still elusive and similarly its role in monocytes is unclear. We described before that monocytes isolated from the blood vessels of renal allografts during fatal acute rejection undergo apoptosis when cultivated ex vivo [43]. As Tgm2 is more strongly expressed in apoptotic cells [11, 13, 44], we investigated if levels of activated caspase-3 correlate with expression of Tgm2 in perfusate cells. Indeed, a stronger induction of activated caspase-3 was seen in leukocytes isolated from day 9 allografts compared to respective isografts. This suggests that Tgm2 expression by blood mononuclear cells might be linked to apoptosis. In line with the idea that Tgm2 enhances apoptosis depending on a prolonged contact with endothelial cells in the inflamed allograft, activation of caspase-3 was more visible in veins compared to arteries. Tgm2 plays a dual role in apoptosis: expression of Tgm2 can induce apoptosis but it also stabilizes the cytoskeleton of dying cells, which prevents cell rupture and release of proinflammatory cytoplasmic components [13, 44–46]. As a large number of monocytes accumulate in allograft vessels, their apoptosis might be a tremendous anti-inflammatory stimulus capable of reverting rejection. This issue certainly deserves further investigation. In the tissue of day 9 and day 42 allografts, increased mRNA expression of Tgm2 also correlated with increased activation of caspase-3.

Immunohistochemistry, however, evidenced that Tgm2 not only is present in the cytoplasm of graft blood monocytes but was also detected in the surrounding blood plasma. Due to rejection-associated vascular damage and the high number of leukocytes accumulating in the blood vessels of allografts, we assume that blood flow slowed down considerably in allografts and that endothelial cells and underlying extracellular matrix are in intensive and prolonged contact with products secreted locally. In the context of hypertension, Tgm2 and other active transglutaminases were shown to contribute to the pathogenesis of vascular remodeling [47–50]. Active transglutaminases were suggested to cross-link extracellular matrix proteins and to result in an increased rigidity of vascular walls, which precedes inward remodeling of arteries [47–50]. In line with this idea, patients suffering from celiac disease, which typically form autoantibodies to Tgm2, are less likely to have a diagnosis of hypertension [51]. It is not known if Tgm2 plays a similar role in the pathogenesis of CAV. We performed animal experiments to investigate this hypothesis and chronically applied biologically meaningful concentrations of cystamine [52], an inhibitor of transglutaminase activity, for 4 weeks after transplantation. However, our hypothesis was not supported. Within 84 days after surgery, allografts developed first hallmarks of CAI, such as IFTA, CAV, and renal dysfunction, irrespective of cystamine treatment. We can, however, not exclude that extracellular transglutaminase activity is involved in both remodeling and re-remodeling of graft arteries as a protective effect of Tgm2 was reported for experimental atherosclerosis [23, 53, 54].

This study has several limitations. The experimental rat model is characterized by a minor mismatch in the MHC class-1 locus and does not include immunosuppression, which is not typical for clinical transplantation. In spite of these disadvantages, it is a favorable, experimental model to investigate an acute rejection episode preceding CAI [9]. We investigate neither transglutaminase activity in the graft nor excretion of its products, and animal experiments were performed using a single dose of transglutaminase inhibitor, which was applied for just one month. We ignore if treatment with other concentrations of cystamine or later time-points would lead to allograft protection. To evaluate the effect of cystamine, we focus on renal function and graft remodeling within 84 days after transplantation. We do not know if, after longer periods of time, differences would have developed among the experimental groups. In addition, cystamine is a weak and rather unspecific inhibitor of Tgm2 as it also inhibits other members of the transglutaminase family as well as certain proteases. More specific inhibitors for Tgm2 are commercially available [55]. Finally, apart from its role in apoptosis, there is still a plethora of potential functions of Tgm2 [11, 15], some of which are enumerated in the introduction, that are awaiting investigation in the context of acute and chronic allograft rejection. Only a part of these functions are inhibited by exogenous cystamine [55].

In conclusion, this is the first study to demonstrate expression of Tgm2 by monocytes activated in vivo during a reversible acute rejection episode. Potentially, the function of monocytic Tgm2 is induction of apoptosis, which in turn might contribute to the reversion of acute rejection in this experimental model. Our data do not support but also do not falsify the hypothesis that Tgm2 expressed by graft monocytes during reversible acute rejection contributes to an early induction of CAI.

## Abbreviations

CAI: Chronic allograft injury
CAV: Chronic allograft vasculopathy
F344: Fischer-344
IFTA: Interstitial fibrosis and tubular atrophy
Tgm2: Transglutaminase 2.
